# Indian research: Focus on clozapine

**DOI:** 10.4103/0019-5545.64592

**Published:** 2010

**Authors:** Sandeep Grover, Alakananda Dutt, Ajit Avasthi

**Affiliations:** Department of Psychiatry, Postgraduate Institute of Medical Education and Research, Chandigarh - 160 012, India

**Keywords:** Clozapine, India, schizophrenia

## Abstract

Clozapine has been used in the treatment of schizophrenia for about two decades and extensive data have been accumulated with regard to its use in various parts of the world. However, in contrast to Western countries, there are few studies which have evaluated the usefulness of clozapine in Indian patients. This article attempts to review the available data on clozapine originating from India. This review reflects the fact that there are few studies from the Indian subcontinent and most of these are case reports. In view of the same, there is a need for further research to evaluate the effectiveness of clozapine in India.

## INTRODUCTION

Clozapine was discovered in 1958 in Bern, Switzerland, and in clinical trials it was shown to be an effective antipsychotic agent that didn’t have extrapyramidal side effects. However, the enthusiasm about clozapine was dampened with the discovery of its hematological toxicity.[1] About 50 patients around the world died due to agranulocytosis and eventually it was withdrawn from most of the European markets in the eighties.[1] However, in 1988, John Kane reported that clozapine was more effective than chlorpromazine.[2] This was followed by other studies and all these efforts led to approval of clozapine in United States in 1990 for patients with schizophrenia who are resistant to treatment with other antipsychotics or who are unable to tolerate conventional antipsychotics because of extrapyramidal side effects or severe tardive dyskinesia (TD).[3]

Over the last two decades extensive data has accumulated with regard to use of clozapine worldwide,[4,5] but only a handful of studies, chiefly in the form of case reports, are from India.

Lately, there was a review in Indian Journal of Psychiatry[6] that focused on clozapine, but there was no reference to Indian data. In this article we review Indian data. The search strategies for this review included both search of electronic databases as well as manual search of relevant publications or cross references. Electronic search included both PUBMED searches and searches using other search engines. Cross-searches of key references (both electronic and manual search) often yielded other relevant material. The search terms used (in various combinations) were: clozapine, India, side effects, efficacy, and effectiveness. Although an attempt was made to include all the articles, however, it cannot be guaranteed that all articles referring to clozapine in various contexts have been included. In the literature search we could find only 17 studies (out of which details of two[7,8] were not available) and 37 case reports/series describing the various aspects of clozapine originating from India.

## STUDIES FOCUSING EFFICACY/EFFECTIVENESS OF CLOZAPINE

Only 9 out of the 17 studies have evaluated the efficacy or effectiveness of clozapine, chiefly focusing on the short-term improvement and three on the long-term effects. In an open-labeled study, Desai *et al*.[9] used clozapine in 28 patients with treatment-resistant schizophrenia (TRS) and found significant improvement in the psychopathology in terms of ‘Brief Psychiatric Rating Scale’ (BPRS) ratings with mean dose of 241 mg/day. They did not observe any side effects in the form of leucopenia or seizures and the most common side effects noticed with clozapine were sedation and sialorrhea in 92.85% of cases. Chand *et al*.[10] described the use of clozapine in an open-labeled study. The authors concluded that the results with clozapine were highly encouraging for the treatment of TRS cases and the common side effects noticed were tachycardia (60%), sedation (33%), and hypersalivation (27%). Agranulocytosis, cardiomyopathy and nephritis were not seen in any case. Mendhekar[11] described the usefulness of high doses of clozapine (>500 mg/day) in 12 TRS cases. They found improvement in psychopathology on PANSS and CGI scale. No case developed hematological side effects and two cases showed seizure activities in EEG and one had drug-induced seizure. Srivastava[12] evaluated 19 patients after three years of starting clozapine. Two patients had been changed to conventional neuroleptic medication at the time of follow-up, and were excluded from the study. Rest of the patients were on monotherapy and the average daily dose was 248.21 mg/day. Significant reduction in psychopathology was observed in 85% of patients. Besides the improvement in psychopathology, patients had improvement in social functioning, with seven patients pursuing a career independently, and another six working with their family members since being started on clozapine. No patient required hospitalization and there was no incidence of granulocytopenia during the 3-year follow-up period. Kale *et al*.[13] evaluated the efficacy of combination of clozapine with ECT in 18 patients diagnosed of schizophrenia, mania, or schizoaffective disorder with no or partial response to medication. The maximum dose of clozapine used was 400 mg/day and on an average six ECTs were used. At the end of the study, all the 18 subjects showed at least 70% improvement on BPRS and 6 of the 18 showed improvement on YMRS. However, the authors did not report the duration of follow-up. In another open-labeled study, Raghuraman *et al*.[14] examined the improvement in psychopathology of 22 cases of treatment-resistant schizophrenia with clozapine therapy after 20 months of starting clozapine. The dose of clozapine used was 300-400 mg/day. During the initial treatment phase moderate side effects in the form of hypersalivation, drowsiness, and anergia were observed, but these subsided within 10-12 weeks. At 20 months follow-up, 50% of the study group responded (25% reduction in the PANSS score from the baseline) to clozapine therapy. A significant reduction of negative symptom and general psychopathology symptom scores of PANSS was also noted, but overall patients with paranoid schizophrenia showed a better response compared to undifferentiated schizophrenia. Besides reduction in psychopathology, patients also showed a better global functioning with clozapine and none of the patients showed deterioration in abnormal involuntary movements, rather, a marginal improvement was noticed in the majority of patients. Although no gender differences were seen in the response rate, significant improvement was seen in the urban population, which authors attributed to good understanding of the illness, family support, and better access to hospital. In a retrospective study, Elsie *et al*.[15] reported usefulness of clozapine in 15 patients with bipolar affective disorder. About one-third of them maintained well for two years after starting of clozapine. More than half (60%) of the subjects received clozapine in the dose of 325–700 mg/day and 80% of the sample had adverse effects in the form of sialorrhea, myoclonic jerks, sedation, and constipation. Forty percent of them were started on prophylactic antiepileptic medications for risk of seizures. However, there was high attrition rate due to cost, poor response, and adverse effects. In another retrospective study, Riswin *et al*.[16] reported usefulness of clozapine in 68 nonbipolar subjects, mostly those with diagnosis of schizophrenia. Three-fourths of them showed good clinical response and more than 75% of them developed clozapine-related side effects. In a recently published study Dutt *et al*[17] evaluated the short term and long term effectiveness of clozapine in 51 subjects (predominantly those diagnosed with schizophrenia) and reported that during the inpatient stay (mean duration 63 days), there was 34.7% reduction in total PANSS rating after starting clozapine at a mean dose of 298.97 mg/day. During the mean duration of follow-up of 3.99 (SD 3.13) years after starting clozapine, of the 51 subjects, 37 cases were still on clozapine at the time of last follow-up. In terms of side effects, sialorrhoea was seen in 58.8% of the patients and other common side effects observed were sedation (47.0%), constipation (15.6%), severe hypotension (2%, N = 1), urinary incontinence (2%) and urinary retention (2%). While receiving clozapine, three cases developed leucopenia (total leukocyte count <4000/cumm) and four patients developed seizures. In two of the three cases, leucopenia led to stoppage of clozapine. Clozapine was stopped only in 1 case due to seizure.

## STUDIES FOCUSING ON SIDE EFFECTS OF CLOZAPINE

Jagadheesan *et al*.[18] in a retrospective study investigated the rate of occurrence, severity, and course of clozapine-induced thrombocytopenia (≤100 × 10^9^/L). Out of 28 patients who received clozapine (duration 21-184 weeks), five (17.8%) had experienced at least one episode of thrombocytopenia. All five patients developed thrombocytopenia while taking therapeutic doses of clozapine ranging from 50-400 mg/day. There was no difference between those who developed thrombocytopenia and those who did not, with respect to age at the time of starting start of clozapine therapy, gender, psychiatric diagnosis, duration of illness, and duration of clozapine therapy. The minimum interval between the start of clozapine therapy and the appearance of thrombocytopenia was 6 weeks and the maximum interval was 44 weeks. The episodes of thrombocytopenia lasted for 1-12 weeks and all the episodes resolved without reducing the clozapine dosage. None of the patients had an overt clinical manifestation or complication of thrombocytopenia or other hematological abnormalities such as leukopenia, neutropenia, or agranulocytosis. Of 4 patients (80%) who had more than one episode of thrombocytopenia, the condition was worse in subsequent episodes for 3 patients. In another study, Praharaj *et al*.[19] used clonidine for clozapine-induced sialorrhea in 12 stable outpatients with schizophrenia maintained on clozapine. At four weeks of treatment most of the patients reported a decrease in sialorrhea without any adverse events.

## STUDY FOCUSING ON COST

A study from Chennai which included 68 persons with schizophrenia assessed the direct cost of treatment with clozapine. Of the 68 patients, 42 were males, and the mean duration of illness was around 12 years. The mean age of the sample was 37 years. Before they were started on clozapine, most of the patients had been on a combination of at least 2-3 neuroleptics as well as some adjuvant drugs. When they were shifted to clozapine, in most cases, it was used as monotherapy and the average dose was about 200 mg per day and no adjuvants were required. After a period of six months, the authors found that despite the costs of blood tests, the total cost of treatment with clozapine came down by nearly 25%. Except for two cases, the families of most of the patients were spending less on treatment. None of these patients had to be hospitalized and all of them continued as outpatients.[20]

## STUDY OF AUGMENTATION OF CLOZAPINE WITH RISPERIDONE

Solanki *et al*.[6] in their review mentioned about their experience of augmentation of 100 clozapine nonresponders with risperidone. They found that augmentation lead to improvement in CGI, BPRS, and PANSS over the period of four weeks.

## SURVEY OF PSYCHIATRISTS ABOUT USAGE OF CLOZAPINE

Shrivastava and Shah[21] surveyed 117 psychiatrists, most of whom had more than 10 years of experience, using a 32-item questionnaire about the usage of clozapine, and reported that clozapine was widely used in patients who do not respond to other available antipsychotics. Forty-three percent of the psychiatrists opined that clozapine provided good or excellent results. Majority of the psychiatrists opined that they prefer to use clozapine as a single agent in TRS cases, and more than half of them initiated clozapine in the dose of 25 mg/day and the dose of 150 to 300 mg/day was considered effective by half of them. Majority of the psychiatrists expressed that clozapine leads to 40 to 70% reduction in the psychopathology. Majority of the psychiatrists preferred blood monitoring every week for initial 6 weeks, than monthly for next 6 months and thereafter as per the need. The common side effects observed by most psychiatrists were sialorrhoea and sedation.

## CASE REPORTS/SERIES FOCUSING ON EFFICACY/USEFULNESS IN VARIOUS FORMS OF PSYCHOSIS

Srinivasan and Latha[22] reported effective and safe use of clozapine in a 9-year-old girl with schizophrenia. Srinivasan[23] used clozapine in the doses of 12.5–50 mg/day in four cases of dementia with severe psychiatric symptoms. All the cases responded immediately to clozapine and the symptoms like insomnia and speaking loudly improved after first dose of clozapine. Most of the other behavioral problems remitted fully in 4–6 weeks. Family members also reported moderate to marked improvement on a visual analog scale. Malhotra *et al*.[24] reported use of clozapine in five cases of childhood-onset schizophrenia. They reported that the level of improvement in positive symptoms was greater than that in negative symptoms with no effect on ritualistic behavior. No serious side-effects (seizures, agranulocytosis) were observed. Raju *et al*.[25] reported improvement with a combination of clozapine and risperidone in two cases of treatment-resistant schizophrenia, who didn’t respond to either clozapine (600–800 mg/day) or risperidone (2–10 mg/day) when used alone. Pradhan and Malhotra[26] reported the usefulness of risperidone in augmentation of efficacy of clozapine in two cases of treatment-refractory schizophrenia. Mendhekar[27] reported use of clozapine in oneriod state (schizophrenia with a clouding of consciousness, occurring mostly in the acute stage of schizophrenia) in a 19-year-old woman. She was treated with clozapine 200 mg/day, with which marked improvement was observed in her psychotic symptoms and confusional behavior.

Dutta and Kumar[28] reported clozapine responsive cluster headache in a 19-year-old girl. Patient had history of headache for eight years and was diagnosed with schizophrenia for the past five years. Her psychosis had not responded to many typical and atypical antipsychotics. After this she was started on clozapine which was increased to 100 mg and after four weeks she stopped having any further headache and her psychotic symptoms had remarkably reduced.

## CASE REPORTS REPORTING SIDE EFFECTS

Agarwal and Khalid[29] described development of obsessive compulsive symptoms in three cases while receiving clozapine. In one case the symptoms were transient (predominantly compulsive), in the second case the symptoms reduced with reduction in dose of clozapine (symptoms were only obsessive doubts), and the third case required stoppage of clozapine due to severity of obsessive symptoms (both obsessions and compulsions). Eranti and Chaturvedi[30] described marked variation in thrombocyte count (both thrombocytopenia and thrombocytosis) without agranulocytosis in a 19-year-old male, while receiving clozapine in the dose range 150–200 mg/day. Kirpekar *et al*.[31] reported reversible myocarditis with clozapine (300 mg/day) after four months of therapy in a 26-year-old patient with chronic resistant schizophrenia. The myocarditis recovered rapidly on withdrawal of clozapine and with supportive management. Kumar *et al*.[32] described development of delirium in a 56-year-old patient with schizophrenia, when clozapine was given to a patient receiving ECT and the dose was increased from 50 to 75 mg/day. However, the patient showed improvement in cognitive functioning, when ECT was stopped and only clozapine was continued and hiked to 350 mg/day. The authors advised caution in use of clozapine along with ECT. Kurian *et al*.[33] described three cases, in which clozapine was used in the dose of 350–450 mg/day along with ECT (8–10 ECTs) without significant cognitive deficits or delirium. Mendhekar and Jiloha[34] described a case of neuroleptic malignant syndrome in a patient with a single dose of haloperidol after abrupt discontinuation of clozapine. The authors attributed the neuroleptic malignant syndrome to dopamine-acetylcholine imbalance following abrupt discontinuation of clozapine. Pillai *et al*.[35] described occurrence of diabetic ketoacidosis in a 45-year-old schizophrenic patient on clozapine, which ultimately led to death in the case. Mendhekar and Duggal[36] described development of tardive dyskinesia (TD) in a patient diagnosed with schizophrenia and hypothyroidism and receiving clozapine 150 mg/day along with thyroid supplement. The TD did not change in frequency and intensity, with change in dose of clozapine to either 125 mg/day or 200 mg/day. Stoppage of thyroid supplement also did not have any impact on the movements. Mendhekar and Duggal[37] described development of oculogyric crisis in a patient with diagnosis of mental retardation with abrupt stoppage of clozapine 300 mg/day. The oculogyric crisis resolved on reinstitution of clozapine in original dose. Thomas *et al*.[38] described a case of cardiac failure in a 73-year-old woman with dementia, who was given clozapine (dose 12.5–100 mg/day) for treatment-resistant delusions. Patient presented with breathlessness within three weeks of starting clozapine and on examination showed signs of cardiac failure with sinus tachycardia and poor R-wave progression in V1-V3. An echocardiogram revealed global left ventricular hypokinesia, mild left ventricular systolic dysfunction, a left ventricular ejection fraction of 40% and moderate mitral regurgitation. Clozapine was stopped and the patient was treated with conventional antifailure measures, with which she improved. Praharaj and Arora[39] described nocturnal enuresis and troublesome sialorrhea with 400 mg/day clozapine in a case of TRS. He was treated with amitriptyline 25 mg at bedtime which led to rapid and complete resolution of enuresis after four days and marked reduction of nocturnal sialorrhea, with disappearance of daytime sialorrhea. Duggal and Mendhekar[40] reported a case of clozapine (100 mg/day) induced tardive dystonia in a 46-year-old woman with schizophreniform disorder after four months of treatment with clozapine. While on clozapine she developed frequent (40 times in a minute) and forceful blinking of her eyelids, which would be exacerbated by bright light. The patient had no prior personal or family history of blepharospasm or other movement disorders. The blepharospasm continued even after four weeks of discontinuing clozapine. Subsequently, she was treated with clonazepam (1 mg/day), with which blepharospasm disappeared after 1 month. Two years later, the blepharospasm reappeared on being rechallenged with clozapine in the dose of 75 mg/day. Mishra *et al*.[41] described two cases of classical angioneurotic edema on clozapine therapy (50–150 mg/day), which recovered rapidly in 2–3 days when clozapine was stopped and the patients were treated with injection dexamethasone/hydrocortisone and pheneramine. Mendhekar and Duggal[42] described double incontinence in a case of 23-year-old male, diagnosed with schizophrenia within 3 weeks of starting clozapine. While he had bowel incontinence almost daily during the afternoon when he was awake, the bladder incontinence was only nocturnal. However, bowel and bladder incontinence never occurred simultaneously. Patient discontinued clozapine due to incontinence and this led to amelioration of the symptoms, but the double incontinence returned on restarting clozapine. Following which clozapine was again discontinued, which led to resolution of double incontinence in 2 days. Subsequently, he was treated with risperidone 2 mg/day without any recurrence of the incontinence. Duggal and Mendhekar[43] reported a case of bipolar affective disorder who developed restless leg syndrome when clozapine was added in the dose of 25 mg/day and increased to 50 mg/day along with valproate 1400 mg/day. On the 3rd day of treatment with clozapine (at 50 mg/d), patient started experiencing unpleasant sensations in his calves and burning sensation over his feet. These symptoms would occur only when he would lie in bed at night and would be relieved by moving the legs or by walking. Initially the symptoms were considered to be a manifestation of akathisia, and he was given lorazepam, but patient did not benefit. Following this clozapine was stopped, with which the symptoms ameliorated. However, the symptoms again recurred following rechallenge with clozapine, following which he was shifted to olanzapine. Raghuraman and Vijaysagar[44] described a case of 28-year-old male, diagnosed with schizophrenia and developed TD of upper limb and orofacial dyskinesia while receiving risperidone 6 mg/day. Due to the movement disorder, he was shifted to clozapine and the dose was titrated to 300 mg/day. Following this his dyskinesia worsened and he was shifted to amisulpride, with which his TD improved. Sagar *et al*.[45] described the case of a 26-year-old male, who was diagnosed with schizophrenia and was receiving clozapine 150 mg/day, who had frequent ventricular premature contractions (VPCs) of left bundle branch block (LBBB) morphology. Multiple gated acquisition (MUGA) scan revealed a mildly dilated left ventricular cavity, with left ventricular ejection fraction of 52%. His clozapine was stopped and he was managed with carvedilol (6.25 mg twice a day), L-carnitine (330 mg three times a day), digoxin (0.25 mg once a day five times/week), and ramipril (started at 2.5 mg/day and increased to 7.5 mg/day). The patient was also treated with antioxidants in the form of selenium, vitamin E, and vitamin A. The patient’s cardiac status gradually began to improve. The MUGA scan done at one year follow-up showed left ventricular ejection fraction of 55%, and the ECG revealed no abnormalities. Recently, Shankar[46] reported a case of delirium, probably arising due to cholinergic imbalance while receiving clozapine 125 mg per day and the delirium subsided on stopping clozapine. Krishnakanth *et al*[47] described development of stuttering in 3 cases while receiving clozapine (200 to 600 mg/day) and in 2 of these 3 cases stuttering led to discontinuation of clozapine.

## CASE REPORTS REPORTING ITS EFFECT ON PREGNANCY AND NEWBORN

Mendhekar *et al*.[48] described the use of clozapine in pregnancy and development of intrauterine growth retardation (IUGR) with oligohydramnious and intrauterine death. Gupta and Grover[49] reported safety of clozapine (100–200 mg/day) in two successive pregnancies in the same patient with no adverse effects on the fetuses. Sethi[50] described the safety of clozapine (250 mg/day) in pregnancy of a 20-year-old female with treatment-resistant schizophrenia. Sethi[50] also reported no developmental delay in the child during the observation period of 2 years. Mendhekar[51] reported delayed speech acquisition in a child, whose mother received clozapine (100 mg/day) throughout the pregnancy and during lactation (breastfed for one year). The baby had normal developmental milestones, except for speech. She began to use consonants at the age of one year and started using combined syllables, like ba-ba and da-da, at the age of one-and-a-half years. At the age of two years she spoke only 6 to 8 words and by the age of 3 years she spoke only 12–15 words. Even intervention by a speech therapist did not lead to improvement. However, by the age of 4 years, she acquired speaking skills in the form of making small sentences by joining two or three words, and by the end of 5 years, she gained normal fluent speech.

## CASE REPORTS FOCUSING ON OVERDOSE OF CLOZAPINE

Mendhekar *et al*.[52] reported clozapine overdose in two cases. The patients had consumed clozapine approximately 10 times higher than their therapeutic dose, and presented to the emergency ward with altered sensorium. On examination, they were found to have tachycardia, hypotension, and respiratory depression. Prompt supportive medical treatment, including administration of intravenous fluids, led to successful recovery without neuropsychiatric sequelae.

## CASE REPORTS FOCUSING ON MANAGEMENT OF CLOZAPINE INDUCED SIDE EFFECTS

Srinivasan and Thomas[53] described a case of young patient who developed total absence of granulocytes during the fourth month of treatment with clozapine and who was successfully treated with G-CSF. Guha and Nizamie[54] described a case of TRS, who was treated with clozapine and developed seizures with clozapine. Addition of sodium valproate to clozapine, led to improvement in schizophrenia symptomatology as well as adequate control of seizures. Aggarwal *et al*[55] described the use of 6 mg/day of trihexyphenidyl for clozapine induced nocturnal enuresis and sialorrhea.

## CASE REPORTS FOCUSING ON USEFULNESS OF CLOZAPINE IN MANAGEMENT OF SIDE EFFECTS OF OTHER PSYCHOTROPICS

Arora *et al*.[56] reported improvement in olanzapine-induced Pisa syndrome in a 22-year-old male with clozapine 350 mg/day in 6 weeks. Chakrabarti and Chand[57] used clozapine (150–200 mg/day) for the treatment of lithium-induced tardive dystonia in a 38-year-old man with bipolar affective disorder. Kumar *et al*.[58] reported a case, in which neurogenic bladder was treated with clozapine.

## CONCLUSIONS

This review shows that most of the studies on clozapine in India are either in the form of open-labeled studies, retrospective chart reviews, or case reports. The total available literature is on about 500 patients only. None of the studies have evaluated clozapine in a randomized controlled trial. Despite this limitation it can be concluded that clozapine leads to significant improvement in patients with schizophrenia having history of poor response to other antipsychotics. There is some evidence in the form of case reports of its usefulness in treatment of conditions like lithium-induced tardive dystonia and neurogenic bladder. Further, serious side effects like seizures and agranulocytopenia are rare. However, there is a need for more well-designed studies from Indian subcontinent to evaluate the usefulness of clozapine.
